# Fast-resorbable antibiotic-loaded hydrogel coating to reduce post-surgical infection after internal osteosynthesis: a multicenter randomized controlled trial

**DOI:** 10.1007/s10195-017-0442-2

**Published:** 2017-02-02

**Authors:** Kostantinos Malizos, Michael Blauth, Adrian Danita, Nicola Capuano, Riccardo Mezzoprete, Nicola Logoluso, Lorenzo Drago, Carlo Luca Romanò

**Affiliations:** 1 Orthopaedic Surgery and Trauma, Medical School, University of Thessaly, Larissa, Greece; 2 Department for Trauma Surgery, Medical University, Innsbruck, Austria; 3 Department for Orthopaedics, San Luca Hospital, Vallo Della Lucania, Italy; 4 Department for Orthopaedics, San Camillo de Lellis Hospital, Rieti, Italy; 5 Department of Reconstructive Surgery of Osteo-articular Infections CRIO Unit, IRCCS Galeazzi Orthopaedic Institute, Via R. Galeazzi 4, 20161 Milan, Italy; 6 Laboratory of Clinical Chemistry and Microbiology, IRCCS Galeazzi Orthopaedic Institute, Milan, Italy; 7 Laboratory of Medical Technical Sciences, Department of Biochemical Sciences for Health, University of Milano, Milan, Italy

**Keywords:** Infection, Prevention, Osteosynthesis, DAC, Hydrogel, Coating

## Abstract

**Background:**

Infection is one of the main reasons for failure of orthopedic implants. Antibacterial coatings may prevent bacterial adhesion and biofilm formation, according to various preclinical studies. The aim of the present study is to report the first clinical trial on an antibiotic-loaded fast-resorbable hydrogel coating (Defensive Antibacterial Coating, DAC^®^) to prevent surgical site infection, in patients undergoing internal osteosynthesis for closed fractures.

**Materials and methods:**

In this multicenter randomized controlled prospective study, a total of 256 patients in five European orthopedic centers who were scheduled to receive osteosynthesis for a closed fracture, were randomly assigned to receive antibiotic-loaded DAC or to a control group (without coating). Pre- and postoperative assessment of laboratory tests, wound healing, clinical scores and X-rays were performed at fixed time intervals.

**Results:**

Overall, 253 patients were available with a mean follow-up of 18.1 ± 4.5 months (range 12–30). On average, wound healing, clinical scores, laboratory tests and radiographic findings did not show any significant difference between the two groups. Six surgical site infections (4.6%) were observed in the control group compared to none in the treated group (*P* < 0.03). No local or systemic side-effects related to the DAC hydrogel product were observed and no detectable interference with bone healing was noted.

**Conclusions:**

The use of a fast-resorbable antibiotic-loaded hydrogel implant coating provides a reduced rate of post-surgical site infections after internal osteosynthesis for closed fractures, without any detectable adverse event or side-effects.

**Level of evidence:**

2.

## Introduction 

The Centers for Disease Control (CDC) healthcare-associated infection (HAI) prevalence survey estimated 157,500 surgical site infections (SSIs) associated with inpatient surgeries in 2011 in the USA [1] (Table 1).

**Table 1:** Criteria for defining a surgical site infection (SSI), according to the CDC criteria (cf. https://www.cdc.gov/hicpac/SSI/table1-SSI.html)

**Superficial incisional SSI** ^*†*^
Infection occurs within 30 days after the operation and infection involves only skin or subcutaneous tissue of the incision and at least one of the following:
Purulent drainage, with or without laboratory confirmation, from the superficial incision
Organisms isolated from an aseptically obtained culture of fluid or tissue from the superficial incision
At least one of the following signs or symptoms of infection: pain or tenderness, localized swelling, redness, or heat and superficial incision is deliberately opened by surgeon, unless incision is culture-negative
Diagnosis of superficial incisional SSI by the surgeon or attending physician
Do not report the following conditions as SSI
Stitch abscess (minimal inflammation and discharge confined to the points of suture penetration)
Infection of an episiotomy or newborn circumcision site
Infected burn wound
Incisional SSI that extends into the fascial and muscle layers (see deep incisional SSI)
**Deep incisional SSI** ^‡^
Infection occurs within 30 days after the operation if no implant† is left in place or within 1 year if implant is in place and the infection appears to be related to the operation and infection involves deep soft tissues (e.g., fascial and muscle layers) of the incision and at least one of the following:
Purulent drainage from the deep incision but not from the organ/space component of the surgical site
A deep incision spontaneously dehisces or is deliberately opened by a surgeon when the patient has at least one of the following signs or symptoms: fever (>38 °C), localized pain, or tenderness, unless site is culture-negative
An abscess or other evidence of infection involving the deep incision is found on direct examination, during reoperation, or by histopathologic or radiologic examination
Diagnosis of a deep incisional SSI by a surgeon or attending physician
**Organ/space SSI** ^‡^
Infection occurs within 30 days after the operation if no implant† is left in place or within 1 year if implant is in place and the infection appears to be related to the operation and infection involves any part of the anatomy (e.g., organs or spaces), other than the incision, which was opened or manipulated during an operation and at least one of the following:
Purulent drainage from a drain that is placed through a stab wound‡ into the organ/space
Organisms isolated from an aseptically obtained culture of fluid or tissue in the organ/space
An abscess or other evidence of infection involving the organ/space that is found on direct examination, during reoperation, or by histopathologic or radiologic examination
Diagnosis of an organ/space SSI by a surgeon or attending physician

^*†*^Report infection that involves both superficial and deep incision sites as deep incisional SSI

^‡^Report an organ/space SSI that drains through the incision as a deep incisional SSI

In spite of improved operating room, sterilization methods, barriers, surgical technique and routine systemic antimicrobial prophylaxis [2–5], SSIs are still considered to be the most common and costly healthcare-associated infection, accounting for 31% of all HAIs among hospitalized patients [6, 7].

After osteosynthesis for closed fractures, early SSI had a reported incidence of 3.9% in a large multicenter trial, with a median time to diagnosis of 30 days [8], while wound healing problems, like those occurring in subcutaneous osteosynthesis [9], and the presence of co-morbidities may increase the risk of septic complications up to 10% [10–12]. In a more recent retrospective study, the rates of infection within 1 year from internal osteosynthesis after closed and open fractures have been reported to be 4.2 and 14.7%, respectively [13]. Implant-related infections often require implant removal, with high morbidity and possible increased mortality [9] and elevated economic and social costs [14].

In this context, antibacterial coatings of implants may represent an attractive option to reduce post-surgical infections [15]. A strong recommendation was delivered in a recent international Consensus meeting on peri-prosthetic joint infections, concerning the need for developing effective antibacterial surfaces that prevent bacterial adhesion and colonization of implants and proliferation into the surrounding tissues [16]. However, only few anti-bacterial coating technologies are currently available in orthopedics and trauma and, for various reasons, they are still far from large-scale application [17, 18].

Developing a new antibacterial coating appears challenging. Since bacterial colonization, from microbial adhesion to an established mature biofilm layer, only takes a few hours [19], any antibacterial protection should act at the exact time of surgery and possibly only for a few hours or days thereafter, to minimize the risk of long-term bacterial resistance induction. Moreover, any new technology has to demonstrate safety and lack of interference with bone healing and should prove to be effective as well as sufficiently easy to manufacture and implement into the current clinical practice. Finally, it should be available at an affordable price, after having passed the scrutiny of the complex regulatory pathway [20]. Biocompatible hydrogels have been shown to be able to deliver pharmacological agents locally and can be designed to meet the desired elution pattern [21]. Recently, a fast-resorbable hydrogel coating that can be loaded intra-operatively with various antibacterials has been developed [22]. Based on the observation that bacterial colonization occurs within the first hours after implant and that short-term systemic prophylaxis is equally effective as long-term to prevent post-surgical infections [23], this coating technology introduced for the first time the concept of ‘short-term local protection’ of the implant. In fact, a short-term local delivery system may meet the requirements needed to win the ‘run to the surface’, while limiting possible long-term unwanted side-effects [24]. This novel fast-resorbable hydrogel coating (Defensive Antibacterial Coating, DAC^®^; Novagenit Srl, Mezzolombardo, Italy) is composed of covalently linked hyaluronan and poly-d,l-lactide and is designed to undergo complete hydrolytic degradation in vivo within 48–72 h as well as being able to completely release a variety of different antibacterials at concentrations ranging from 2−10%. The hydrogel showed synergistic antibacterial and antibiofilm activity with various antibiotics and antibiofilm agents in vitro [25], while in vivo it has been proven effective in a rabbit model of highly contaminated implant both with [26] and without systemic prophylaxis, without interfering with bone growth [27]. Following previous brief reports [28, 29], we present the clinical results of a multicenter European trial comparing the SSI rate between patients treated with DAC hydrogel-coated osteosynthesis implants and patients treated with non-coated implants.

## Materials and methods

**Fig. 1:**
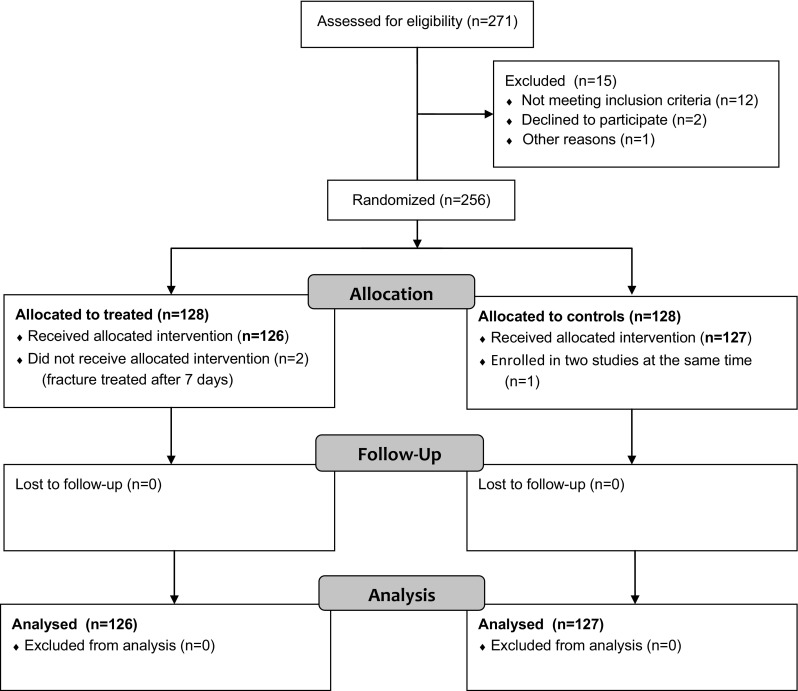
‘Consort flow diagram’ of enrolled patients

From January 2014 to June 2015, 256 patients (Fig. 1) were included in this prospective multicenter randomized study. The study protocol was approved by the local Ethical Committees of the five participating centers. All patients gave their informed consent to the procedure. The study was performed within the 7th European Framework Programme (project #277988) and funded by the European Commission and the participating partners (clinical institutions and the following private companies: Novagenit SRL, Mezzolombardo, Italy, acting as project leader; AdlerOrtho SRL, Bologna, Italy; Arcos SARL, Brignoles, France; Belgafix SPRL, Drogenbos, Belgium).

The patients, in five European orthopedic centers, were randomly assigned through electronic software to receive antibiotic-loaded DAC or to a control group (without coating).

Inclusion criteria were the presence of a fresh (<7 days) closed fracture requiring surgical reduction and internal fixation with either a metal plate and/or screws or with an intramedullary nail, in patients aged >18 years. Exclusion criteria were pregnancy, breast-feeding or planning to become pregnant during the study, the presence of a previous or active infection at site of fracture, severe malignancies with a life expectancy of <3 months, previous diagnosis of immune depression (including HIV) or immune suppressive treatment for organ transplantation, known allergy to the antibiotics or to DAC hydrogel constituents, patient not willing or not able to present for the follow-up consultations or if the patient did not sign the informed consent documents or was not able to do so.

### Surgical treatment and DAC preparation

After routine preoperative work-out, all patients were treated according to the current principles of fracture reduction and internal osteosynthesis. The choice of the surgical approach and the type of osteosynthesis was left to each participating surgeon.

Systemic antibiotic prophylaxis was performed with perioperative administration of a single dose of the antibiotic chosen at each center [30]. All patients also received low-weight heparin for deep vein thrombosis prophylaxis starting on the day of surgery and for 4–6 weeks postoperatively.

Allowed fixation materials included plating, screw and intramedullary nailing systems from Stryker Inc. (New York, USA), Smith-Nephew (London, UK) and DePuy-Synthes (Warsaw, IN, USA), respectively.

Reconstitution of the DAC hydrogel was performed according to the manufacturer’s instructions. Briefly, the prefilled syringe, containing 300 mg sterile DAC powder, was filled at surgery with a solution of 5 mL sterile water for injection and the desired antibiotic. This allowed the antibiotic-loaded hydrogel with a DAC concentration of 6% (w/v) and an antibiotic concentration ranging from 20−50 mg/mL to be prepared in ~3–5 min, depending on the choice of the surgeon. The surgeons could choose the antibiotic from a list of antibacterials previously tested as being compatible with the hydrogel, including gentamicin, vancomycin, daptomycin, meropenem, rifampicin, and ciprofloxacin [25] (Novagenit SRL, data on file).

**Fig. 2:**
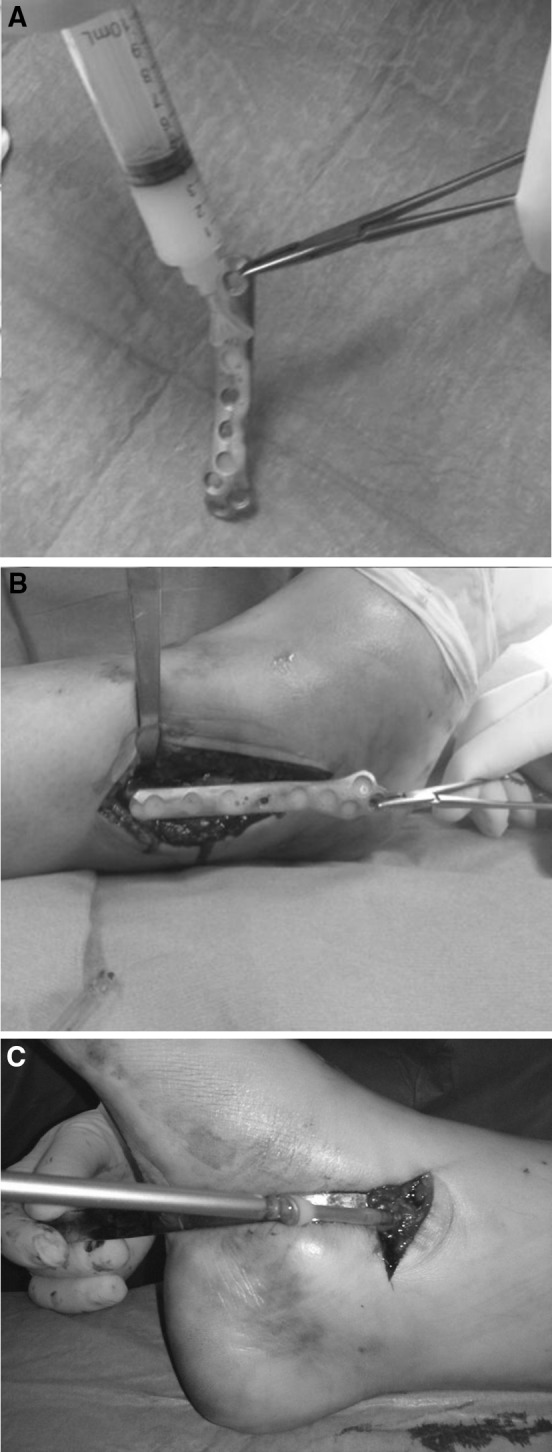
The ‘Defensive Antibacterial Coating’ (DAC^®^) hydrogel coating is spread onto a plate and a screw for osteosynthesis in an ankle fracture

According to previous studies showing the ability of the hydrogel to resist press-fit insertion [25–27], the hydrogel was directly spread onto the implant surface prior to its insertion into the body, a few minutes after reconstitution. Further hydrogel was eventually applied on the synthesis after its positioning on the bone and at the bone−synthesis interface, in order to achieve complete coverage of the implant surface. Similarly, the hydrogel was applied directly on each pre-drilled screw hole and directly on the screws, at the time of their insertion (Fig. 2). A similar technique was used for coating intramedullary nails and locking screws.

### Assessments

All patients underwent preoperative clinical and radiographic examinations and laboratory tests. Host type was classified according to McPherson et al. [31]. Clinical evaluations, serum laboratory tests and radiographic examinations were also scheduled at 6 ± 4 weeks, 3 months ± 4 weeks, and at 6, 12, 18 and 24 months ± 8 weeks postoperatively.

The primary outcome of the study was the reduction of SSI at a minimum 12-month follow-up in the treated versus the control group. SSI was defined as the presence of positive local clinical signs of inflammation, including pain, redness, warmth, swelling, draining wound, fistulas, etc., according to the CDC procedure-associated module SSI (https://www.cdc.gov/hicpac/SSI/table1-SSI.html) (Table 1), requiring unplanned antibiotic treatment and/or surgery, e.g., early synthesis removal or debridement, with or without a positive cultural examination.

Secondary outcomes were the absence of adverse events and side-effects related to the hydrogel coating, as assessed by clinical, laboratory and radiographic examinations.

To this aim, clinical evaluation was performed using the SF-12 score at follow-up, while serious adverse events and any complication or side-effects were recorded whenever necessary at follow-up. Wound healing was assessed at 7 and 14 days using the ASEPSIS score, described by Wilson et al. [32], while delayed wound healing was defined as incomplete healing of the wound after 4 weeks from surgery, including the presence of wound dehiscence, necrosis or serum leakage that may need further medication but did not require any additional surgical treatment.

Laboratory tests, including erythrocyte sedimentation rate, C-reactive protein, hemocromocytometric, and liver and kidney function markers, were performed at follow-up until 6 months after surgery and whenever SSI was suspected.

Radiographic examination was performed by an independent radiologist not aware of the DAC treatment. Bone healing was defined as the presence of visible bridging between two cortices, while delayed union was defined as a lack of bone healing 6 months after trauma. A non-union was identified when a period of 9 months had elapsed with no healing progress for 3 months.

### Sample size calculation

The primary outcome of this trial was the rate of SSI at a minimum of 12 months postoperation, defined as reported above.

Two hundred and fifty-six patients listed for osteosynthesis of fresh fractures were recruited to the intervention arm or to the standard care arm. Assuming an average expected rate of SSI after osteosynthesis of 6.0% in the control group [8, 9, 13] and an SSI rate of 0.1% in the treated group, a sample size of 122 patients in each arm is sufficient to detect a clinically important difference between the two groups with 80% power and 5% level of significance, as calculated using a two-tailed *z* test of proportions [33]. This significant expected effect size is based upon the rate of post-surgical infection previously investigated in animal models of implant-related infection, using the DAC device [26]. The sample size of 256 patients takes into account an expected drop-out rate of ~9%.

### Statistical analysis

In order to detect a reduction in the rate of deep SSI from 6.0 to 1.0% for a two-sided 5% level of significance and 80% power, for the selected binary outcome we needed a total of 244 participants, assuming a chi-squared test as the definitive analysis.

Baseline demographic and comorbidity data were summarized to check comparability between treatment arms. To assess whether there was any evidence of systematic imbalance introduced by the randomization procedure, we also undertook formal statistical testing of differences in baseline characteristics between treatment arms using independent samples *t* tests and Fisher’s exact test or chi-squared tests, with significance set at the 5% level.

Differences between the groups for other secondary outcomes, including clinical and laboratory tests and complications were assessed using chi-squared and Fisher’s exact tests as appropriate.

## Results

Overall 253 patients (126 treated and 127 controls) were available with an average follow-up of 18.1 ± 4.5 months (range 12–30) and were considered for further analysis (Fig. 1).

The two groups did not differ significantly regarding age, sex and host type. In particular, approximately half of the patients in both groups presented with one or more relevant co-morbidities known to increase post-surgical infection risk (Table 2).

**Table 2:** Demographic and preoperative data of the patients included in the study

	Controls	%	Treated	%	*P*
Male	57	44.9	53	42.1	0.70
Female	70	55.1	73	57.9	
Total	127	100.0	126	100.0	
Age (years)					
Mean ± SD	58.6 ± 17.6		62.5 ± 21.2		0.11
Min–max	20–95		21–99		
Host type					
A	70	55.1	60	47.6	0.25
B	53	41.7	61	48.4	
C	4	3.1	5	4.0	
Fracture site					
Femur	32	25.2	47	37.3	
Tibia/knee	11	8.7	16	12.7	
Ankle/foot	29	22.8	32	25.4	
Clavicle	11	8.7	10	7.9	
Humerus	8	6.3	6	4.8	
Forearm/wrist	29	22.8	14	11.1	
Hand	7	5.5	1	0.8	

Host type classified according to McPherson’s classification

Perioperative data (Table 3) show that the majority of patients were treated with plate/screws and <10% in both groups underwent nail fixation.

**Table 3:** Perioperative data

	Controls	%	Treated	%
Type of fixation				
Plate/screws	117	92.1	115	91.3
Intramedullary nail	10	7.9	11	8.7
Systemic prophylaxis				
Cefazolin	70	55.1	69	54.8
Cefazolin + amikacin	37	29.1	31	24.6
Cefazolin + vancomicin	20	15.7	26	20.6
DAC volume (mL)				
Mean ± SD	N/A		5.7 ± 3.0	
Min–max	N/A		1–10	
DAC + gentamicin	N/A		78	61.9
DAC + vancomycin	N/A		46	36.5
DAC + vancomicin + meropenem	N/A		2	1.6

Cefazolin was the most used antibiotic for short-term systemic prophylaxis in both groups, either alone or in association with amikacin or vancomycin.

On average, 5.7 mL (range 1–10 mL) of DAC hydrogel was needed to coat the implant. Gentamicin and vancomycin were the most used antibiotics, at concentrations of 4 or 2%, respectively.

Early wound healing did not show any difference between groups, with an average ASEPSIS score at 7 and 14 days of 1.53 ± 3.94 and 1.93 ± 5.09 in the control group and 1.51 ± 4.14 and 1.33 ± 4.32 in the treated group, respectively. Delayed wound healing occurred in 7 (5.5%) and 5 (3.9%) in the control and treated group, respectively.

Unplanned antibiotic treatment during hospital stay, for reasons other than SSI (mainly urinary or respiratory tract infections), was reported in 12 (9.4%) and 10 (8%) patients in the control and treated groups, respectively (*P* = 0.8).

At 6 months, average serum laboratory tests (hematological, renal and hepatic function) did not show any significant difference between groups (Table 4).

**Table 4:** Serum laboratory tests at 6 months post-surgery

	Controls (mean ± SD)	Treated (mean ± SD)	*P*
Erythrocyte sedimentation rate (mm/h)	14.3 ± 16	17.3 ± 17	0.32
C-reactive protein (mg/L)	4.1 ± 8.3	4.2 ± 4.6	0.93
Hemoglobin (g/100 mL)	14.6 ± 1.2	14.3 ± 1.7	0.26
White blood cells (cells/mL)	7538 ± 2079	7352 ± 1452	0.57
PMN (%)	59.2 ± 8.5	59.5 ± 7.8	0.84
Creatinine (mg/dL)	0.9 ± 0.19	0.88 ± 0.17	0.54
SGOT (U/L)	21 ± 14.6	22.8 ± 15.5	0.34
SGPT (U/L)	20.4 ± 20.7	23.9 ± 15.5	0.12
GAMMA-GT (U/L)	35.9 ± 33.5	42.5 ± 55	0.24

*PMN* polymorphonuclear leukocytes; *SGOT* Serum Glutamic Oxaloacetic Transaminase; *SGPT* Serum Glutamic Pyruvic Transaminase; *GAMMA-GT* Gamma-Glutamyl Transferase

At an average 12-month follow-up, average SF-12 clinical score did not differ significantly between groups (Table 5).

**Table 5:** Postoperative data at the latest follow-up

	Controls (*N* = 127)	%	Treated (*N* = 126)	%	*P*
Follow-up (months)					
Mean ± SD	18.1 ± 5.2		18.1 ± 3.5		1.0
Min–max	12–30		12–26		
SF-12-physical score					
Mean ± SD	46 ± 11.8		49.3 ± 9.7		
SF-12-mental score					
Mean ± SD	54.4 ± 9.5		52.4 ± 10.6		
SF-12-total score					
Mean ± SD	101.7 ± 15.4		100.5 ± 14.2		0.51
Complications					
Surgical site infection	6	4.7	0	0.0	0.03
Delayed wound healing	7	5.5	5	3.9	0.76
Delayed union	5	3.9	2	1.6	0.44

Delayed union was observed in 5 (3.9%) patients in the control group, compared to 2 (1.6%) in the treated group (*P* = 0.4).

No adverse events attributable to the DAC hydrogel were reported. No detectable interaction was observed between the hydrogel and bone healing. Six SSIs were reported in the control group (4.7%), compared to none in the treated group (*P* = 0.03). One patient in the control group underwent early plate removal for plate intolerance, without reported signs of infection. Detailed information regarding septic complications, including treatment and outcomes are provided in Table 6.

**Table 6:** Data from patients with surgical site infection

Patient no.	Age	Sex	Host type	Relevant co-morbidities	Diagnosis	Type of osteosynthesis	Onset of infection (months from surgery)	Cultural examination	Other complications	Treatment	Final outcome
1	47	M	A	None	Weber C fibula fracture	Plate and screws	1	Negative	Delayed wound healing	Prolonged antibiotic treatment	No infection recurrence
2	39	M	B	Nicotine abuse	Clavicula fracture	Plate and screws	1	Negative	Delayed wound healing	Early plate removal and debridement	No infection recurrence
3	29	M	C	Nicotine and alcohol abuse	Ankle fracture	Plate and screws	2	*Corynebacterium* spp.	Delayed wound healing	Early plate removal and debridement	No infection recurrence
4	88	F	C	Diabetes, peripheral vasculopathy, old age	Proximal femur fracture	Plate and screws	6	*Staph. epidermidis*		Early plate removal and debridement	Infection persistence
5	90	F	B	Diabetes, old age	Proximal femur fracture	Plate and screws	6	*Escherichia coli*		Early plate removal and debridement	No infection recurrence
6	75	F	C	Severe rheumatoid arthritis, old age, corticosteroid therapy	Peri-prosthetic supracondilar femoral fracture	Plate and screws	2	MRSA	Delayed-union	Plate removal and two-stage knee revision prosthesis	No infection recurrence

## Discussion

This is the first clinical trial reporting on the efficacy and safety of DAC coating for internal osteosynthesis.

Concerning efficacy, this study shows that the studied antibiotic-loaded hydrogel coating is able to significantly reduce early SSIs after osteosynthesis, at an average 18-month follow-up. This finding is in agreement with earlier in vivo studies [26, 27] and with a recently published multicenter clinical trial on the use of DAC coating in total hip and knee cementless or hybrid total joint replacement [34]. It is also the first clinical demonstration that short-term local prophylaxis may significantly reduce post-surgical septic complications in internal osteosynthesis for closed fractures.

Clinical demonstration of safety is a basic requirement of any novel coating technology [18, 35]. The reported combined data from five European centers indicate that the device under study can be considered clinically safe, when used in combination with internal osteosynthesis, without any detectable local side-effects both concerning wound and bone healing, at a medium-term follow-up. Moreover, no changes in organ-specific serum markers or systemic unwanted effects were recorded. This finding is in line with previous data from in vivo and clinical studies [26, 27, 34]. The high biocompatibility of its basic constituents and the short time (<3 days) needed for complete hydrogel resorption [22, 25] make the possible occurrence of longer term side-effects unlikely.

In isolated reports, antibacterial coatings have previously been shown to be clinically effective in reducing septic complications; however their application to osteosynthesis is limited [18, 28].

Silver coating is among the most extensively studied antibacterial agents. Dissolved silver ions are biochemically active agents, able to interfere with bacterial cell membrane permeability and cellular metabolism. Silver also contributes to the formation of reactive oxygen species and to other mechanisms that potentially influence prokaryotic cells [36]. There has been concern, however, about the toxicity of silver ions [37] and to overcome this issue, research efforts have recently focused on new silver-coating technologies, that are reported to reduce or even eliminate toxicity while maintaining antibacterial effects [38, 39]. However, despite a demonstrated clinical efficacy and safety in two comparative studies on a limited series of patients treated with oncological endoprosthesis [40, 41], the routine use of silver-coated implants remains limited while, to the best of our knowledge, its application to fracture fixation devices has never been investigated.

A different approach, consisting of the local administration of antibiotics in order to protect an implant, historically attracted much attention in orthopedics. Buchholz et al. first popularized the incorporation of antibiotics into polymethylmethacrylate (PMMA) bone cement for local antibiotic prophylaxis in cemented total joint arthroplasty [42] and, although the use of antibiotic-loaded PMMA coating of nails is gaining increasing interest to treat osteomyelitis, septic non-unions and contaminated fractures [43], comparative clinical studies are lacking. Moreover, PMMA may not be used as a coating for plate osteosynthesis or screws and antibiotic-loaded PMMA may not overcome biofilm formation and has been found to be associated with the development of antibiotic-resistant ‘small-colony variants’ [44, 45].

Other porous biodegradable materials for local antibiotic delivery, like collagen sponges [46], cancellous bone [47], calcium phosphate [48, 49] and bioceramics [50], were not specifically designed to protect implanted biomaterials and their use for infection prevention in trauma is currently limited.

Biodegradable polymers and sol–gel coatings have also been investigated to provide a controlled antibiotic release on titanium [51, 52] or hydroxyapatite implants [53]. However, the most known clinical applications of this approach are probably antibiotic-loaded d-poly-lactate acid/gentamycin intramedullary coated nails that, until now, only showed some positive results in a limited series of patients [17].

In this setting, an antibiotic-loaded fast-resorbable hydrogel coating may offer ease of use, versatility and large scale applications, opening the way to an affordable wide application of antibacterial implant protection, as recently shown in a multicenter trial focused on infection prevention in total hip and knee replacement [34].

This study has some limitations. First, the follow-up is relatively short. Although the minimum 12-month monitoring appears adequate to detect early post-surgical septic complications, exceeding that defined in the IDSA Guidelines [54], and appears adequate to detect the vast majority of SSIs after osteosynthesis [8, 9], longer follow-up could be useful to further investigate the ability of the tested device to eventually prevent the occurrence of delayed and late infections. Second, the designed study deliberately left the participating centers free to choose the systemic antibiotic used for prophylaxis, as well as the one added to the hydrogel locally. To our knowledge, as there is no clear evidence showing the superiority of one antibiotic prophylaxis over another [55], it was decided to leave each center free to decide the prophylaxis on the basis of their experience and the regional microbiology, instead of imposing a fixed arbitrary regimen. Moreover, the main activity of the DAC hydrogel is thought to be its anti-adhesive effect, as recently reported [56], while the presence of the antibiotic in the hydrogel is intended to eventually kill the remaining planktonic bacteria and is ancillary to the main activity of the device. All things considered, the choice to leave the centers free to choose the type of antibiotic did finally provide homogeneous and comparable data and may actually better simulate the real-life possible clinical scenario once the DAC device will be available to market. Other limitations of the study concern the exclusion of exposed fractures or other potentially challenging clinical situations, in which an antibacterial coating could eventually be useful. This will be the object of further planned studies.
